# Clinical Correlates of Cardiovascular Autonomic Measures in Patients with Type 2 Diabetes: A Cross-Sectional Study

**DOI:** 10.3390/jcm15208021

**Published:** 2026-10-16

**Authors:** Alexandra Gogan, Corina Vernic, Ovidiu Potre, Minodora Andor, Florina Rozina Caruntu, Samuel Ardelean, Bogdan Timar

**Affiliations:** 1 Doctoral School of Medicine, “Victor Babes” University of Medicine and Pharmacy, 300041 Timisoara, Romania; alexandra.gogan@umft.ro (A.G.); samuel.ardelean@umft.ro (S.A.); 2 First Department of Internal Medicine, Medical Semiology II, “Victor Babes” University of Medicine and Pharmacy, 300041 Timisoara, Romania; andor.minodora@umft.ro (M.A.); caruntu.florina@umft.ro (F.R.C.); 3 Cardiology Clinic, Institute of Cardiovascular Disease, 300310 Timisoara, Romania; 4 Department III Functional Sciences, Discipline of Medical Informatics and Biostatistics, “Victor Babes” University of Medicine and Pharmacy, Eftimie Murgu Square No. 2, 300041 Timisoara, Romania; 5 First Department of Internal Medicine, Hematology, “Victor Babes” University of Medicine and Pharmacy, 300041 Timisoara, Romania; potre.ovidiu@umft.ro; 6 Multidisciplinary Research Centre for Malignant Hematological Disease (CCMHM), “Victor Babes” University of Medicine and Pharmacy, 300041 Timisoara, Romania; 7 Multidisciplinary Heart Research Center, “Victor Babes” University of Medicine and Pharmacy, 300041 Timisoara, Romania; 8 Cardiology Clinic of Timisoara Municipal Clinical Emergency Hospital, 300040 Timisoara, Romania; 9 Centre for Molecular Research in Nephrology and Vascular Disease, “Victor Babes” University of Medicine and Pharmacy, 300041 Timisoara, Romania; bogdan.timar@umft.ro; 10 Department of Diabetes, “Pius Brinzeu” Emergency Hospital, 300736 Timisoara, Romania; 11 Second Department of Internal Medicine, “Victor Babes” University of Medicine and Pharmacy, 300041 Timisoara, Romania

**Keywords:** diabetes mellitus, type 2 diabetes, chronic complications, cardiac autonomic neuropathy, orthostatic hypotension, QTc interval, cardiovagal function

## Abstract

**Background/Objectives:** Cardiovascular autonomic dysfunction in type 2 Diabetes Mellitus (T2DM) is heterogeneous, and individual autonomic measures may identify distinct clinical profiles. This study assessed the clinical, neuropathic, metabolic, and vascular correlates of three cardiovascular autonomic measures: the expiratory-to-inspiratory (E:I) R–R interval ratio, orthostatic blood pressure response, and corrected QT interval (QTc). **Methods:** This secondary cross-sectional analysis included 109 patients with T2DM from a previously characterized cohort. Cardiovagal function was assessed using the E:I R–R interval ratio during deep breathing. Orthostatic hypotension (OH) was defined according to systolic and diastolic blood pressure changes after standing, while QTc was calculated using Bazett’s formula. Peripheral neuropathy was evaluated using the Michigan Neuropathy Screening Instrument (MNSI) and Toronto Clinical Neuropathy Score (TCNS), and peripheral vascular status using the ankle–brachial pressure index (ABI). Group comparisons, chi-square or Fisher exact tests as appropriate, odds ratios (OR) with 95% confidence intervals (CI), Spearman correlation analyses, and multivariable linear regression analyses were performed. Exploratory families of comparisons and correlations were controlled using the Benjamini–Hochberg false discovery rate procedure. **Results:** A pathological E:I R–R interval ratio was identified in 90 patients (82.6%), prolonged QTc in 23 (21.1%), and orthostatic hypotension in 40 (36.7%). Categorical OH was not associated with the evaluated clinical, neuropathic, or vascular parameters. In contrast, continuous systolic blood pressure change after standing (ΔSBP) was independently associated with peripheral neuropathy severity, assessed using the MNSI and TCNS. The nominal univariable association between ΔSBP and HbA1c did not remain significant after false-discovery-rate correction or multivariable adjustment. QTc prolongation showed no consistent association pattern with the other evaluated autonomic-related measures. **Conclusions:** The evaluated cardiovascular autonomic measures showed partially distinct cross-sectional association patterns in patients with T2DM. Continuous ΔSBP was independently associated with peripheral neuropathy severity, whereas categorical orthostatic hypotension showed no significant associations. The E:I R–R interval ratio and QTc prolongation showed limited overlap with the other evaluated measures. These findings are hypothesis-generating and do not establish the diagnostic utility of individual measures for cardiovascular autonomic neuropathy.

## 1. Introduction

Cardiac autonomic neuropathy (CAN) is a common but frequently underrecognized complication of Diabetes Mellitus (DM) [1]. As diabetes becomes a global epidemic, the incidence of CAN is predicted to rise. In 2019, DM affected 463 million persons globally. This number is projected to rise more than 592 million by 2035, according to the International Diabetes Federation, and to 700 million by 2045 [2,3]. The frequency of CAN varies greatly depending on the method of diagnosis used, the stage of disease, and the population examined. According to significant research, the number of patients with CAN ranges from 17% to 90% in patients with type 1 Diabetes Mellitus (T1DM), and 27.5% to 73% in patients with type 2 Diabetes Mellitus (T2DM) [4]. Because it is linked to potentially fatal clinical conditions like silent myocardial ischemia, coronary artery disease, peri-operative cardiovascular instability, QT interval prolongation, and stroke, which increase mortality, CAN is thought to be the most significant manifestation of diabetic autonomic neuropathy [5].

CAN is defined by progressive loss of parasympathetic and sympathetic innervation of the cardiovascular system. In most patients, parasympathetic dysfunction generally occurs earlier in the course of CAN and is reflected by abnormalities in heart rate response to physiological stimuli, whereas sympathetic involvement becomes more prominent in the later stages of the disease, contributing to abnormalities in vascular regulation and blood pressure (BP) control [6,7,8].

Both sympathetic and vagal function should be tested as part of the diagnostic evaluation of CAN. Cardiac autonomic reflex tests (CARTs) are considered the reference standard for diagnosing CAN. The classical test battery includes heart rate responses to deep breathing, standing, and the Valsalva maneuver, together with blood pressure responses to standing and sustained handgrip. These tests assess complementary aspects of parasympathetic and sympathetic cardiovascular autonomic function [9].

Although cardiovascular autonomic reflex tests are used to diagnose CAN, individual autonomic measures reflect different aspects of cardiovascular autonomic regulation. However, the clinical characteristics associated with abnormalities in these individual measures remain incompletely understood. In particular, it is unclear whether impaired heart rate response to deep breathing, altered orthostatic blood pressure regulation, and QTc prolongation are associated with distinct clinical profiles in patients with T2DM.

A better understanding of the clinical features associated with specific cardiovascular autonomic abnormalities may improve the interpretation of autonomic testing and provide further insights into the various presentations of diabetic autonomic neuropathy. Rather than considering CAN as a single clinical entity, exploring individual autonomic measures may help identify distinct patterns of autonomic involvement and their relationships with peripheral neuropathic, metabolic, and vascular abnormalities. Although cardiovascular autonomic reflex tests are used to diagnose CAN, individual measures reflect distinct aspects of cardiovascular autonomic regulation and should not be interpreted as independently diagnostic of CAN.

A previous publication from this cohort focused primarily on SUDOSCAN-derived electrochemical skin conductance (ESC) and sudomotor dysfunction. That report described the baseline cohort characteristics, comparisons according to overall, hand, and foot sudomotor dysfunction, and the associations of ESC with E:I R–R ratio, orthostatic blood pressure changes, and QTc [10]. The present prespecified secondary analysis addresses a different question: the clinical association patterns of individual cardiovascular autonomic-related measures. It examines the E:I R–R interval ratio, orthostatic blood pressure response, and QTc as separate measures in relation to clinical, metabolic, neuropathic, renal, and vascular characteristics.

## 2. Materials and Methods

### 2.1. Study Design and Participants

The present study is a secondary cross-sectional analysis of a previously characterized cohort of 109 patients with T2DM [10]. All participants were adult inpatients, consecutively enrolled during routine hospital admissions for clinical assessment and treatment optimization. No outpatient recruitment was performed. Participants were evaluated at the Department of Diabetes, Nutrition and Metabolic Diseases of the “Pius Brînzeu” Emergency Clinical County Hospital, Timișoara, Romania, between September and December 2024. The recruitment procedure, eligibility criteria, and original study protocol have been reported previously [10].

The present analysis was prespecified in the original study protocol. The previous publication evaluated SUDOSCAN (Impeto Medical, Paris, France)-derived ESC as the primary measure and reported baseline characteristics, sudomotor group comparisons, associations of ESC with E:I R–R ratio, orthostatic blood pressure changes, and QTc, as well as the discriminatory performance of ESC for these measures. The current analysis focuses on the clinical correlates of the individual E:I R–R interval ratio, orthostatic blood pressure response, and QTc measures.

The study was conducted in accordance with the Declaration of Helsinki. Approval was obtained from the Ethics Committee of the “Victor Babeș” University of Medicine and Pharmacy, Timișoara (No. 45/10 September 2024), and the Local Ethics Committee of the “Pius Brînzeu” Emergency Clinical County Hospital (No. 410/27 October 2023). All participants provided written informed consent before enrollment.

### 2.2. Cardiovascular Autonomic Assessment

For the purpose of the present analysis, cardiovascular autonomic function was characterized through three individual components: the heart rate response to deep breathing, the blood pressure response to standing, and the corrected QT (QTc) interval. The E:I R–R interval ratio was considered a measure of cardiovagal function, the orthostatic blood pressure response a measure of sympathetic vasomotor regulation, and QTc an indirect and non-specific repolarization marker. These measures were examined separately to investigate whether different autonomic abnormalities were associated with distinct clinical, metabolic, neuropathic, and vascular characteristics.

Autonomic assessments were performed in the afternoon, in the fasting state, after at least 5 min of supine rest. Participants were instructed to avoid caffeine intake, smoking, and physical activity before testing. Alcohol consumption did not occur during hospitalization. Patients with atrial fibrillation or another non-sinus rhythm, pacemaker rhythm, bundle branch block or other major conduction abnormalities, and acute medical illness at admission were excluded.

Cardiovagal function was assessed from the heart rate response to deep breathing at six respiratory cycles per minute. The expiratory-to-inspiratory R–R interval ratio (E:I R–R ratio) was calculated by dividing the longest R–R interval during expiration by the shortest R–R interval during inspiration. An E:I R–R interval ratio < 1.10 was considered pathological. This single threshold was prespecified in the original study protocol and was applied uniformly to all participants. Age-adjusted normative reference values were not used.

The cardiovascular response to postural change was assessed by measuring systolic blood pressure (SBP) and diastolic blood pressure (DBP) before and after standing. Orthostatic hypotension was defined as a reduction in SBP of ≥20 mmHg and/or DBP of ≥10 mmHg. A single SBP and DBP measurement was obtained with a manual sphygmomanometer after at least 5 min of supine rest and repeated 1 min after active standing. ΔSBP and ΔDBP were calculated as standing blood pressure minus supine blood pressure; thus, negative values indicate a blood pressure decrease after standing. Symptoms during standing were not systematically recorded.

The QT interval was measured manually on printed resting 12-lead electrocardiograms, from the onset of the QRS complex to the end of the T wave. Lead II was used preferentially; V5 or V6 was used when the T-wave end was more clearly identifiable. U waves were not included in the QT measurement. QT intervals were corrected for heart rate using Bazett’s formula (QTc = QT/√RR). QTc was considered prolonged at values > 440 ms in men and >460 ms in women.

### 2.3. Peripheral Neuropathy Assessment

Peripheral neuropathy was assessed as previously described for this cohort [10]. Briefly, peripheral neuropathic involvement was evaluated using the Michigan Neuropathy Screening Instrument (MNSI) and the Toronto Clinical Neuropathy Score (TCNS), with higher scores indicating greater neuropathy severity.

Peripheral vascular status was evaluated using the ankle–brachial pressure index (ABPI), measured with an automated oscillometric device (MESI ABPI MD; MESI, Ltd., Ljublijana, Slovenia). An ABPI < 0.90 was considered indicative of peripheral arterial disease.

A previous publication from this cohort reported the peripheral and sudomotor neuropathy assessment, including SUDOSCAN (Impeto Medical, Paris, France)-derived electrochemical skin conductance results. Those analyses are not repeated in the present manuscript.

### 2.4. Clinical Laboratory and Vascular Evaluation

Demographic characteristics, diabetes duration, previous cardiovascular events, diabetes-related complications, and glucose-lowering treatment were obtained from the medical records and verified during clinical assessment.

Clinical and laboratory data were obtained as part of the original study protocol [10]. Variables considered in the present analysis included age, diabetes duration, body mass index (BMI), glycated hemoglobin (HbA1c), estimated glomerular filtration rate (eGFR), diabetic retinopathy, and previous cardiovascular events. eGFR was calculated using the CKD-EPI equation. Detailed cardiovascular, antihypertensive, and other medication use with potential effects on heart rate, orthostatic blood pressure, or QTc was not systematically recorded in the original protocol and therefore could not be included as a covariate.

### 2.5. Statistical Analysis

Statistical analyses were performed using MedCalc Statistical Software, version 22.015 (MedCalc Software Ltd., Ostend, Belgium). The distribution of continuous variables was evaluated using the Shapiro–Wilk test. Continuous variables were presented as mean ± standard deviation when normally distributed and as median [interquartile range] when non-normally distributed. Comparisons between independent groups were performed using the Mann–Whitney U test. Associations between categorical variables were evaluated using Pearson’s chi-square test when cell counts were adequate and Fisher’s exact test when any observed cell count was <5. Odds ratios (ORs) and corresponding 95% confidence intervals (CIs) were calculated from 2 × 2 contingency tables. Spearman’s rank correlation analysis was used to investigate relationships of the continuous E:I R–R interval ratio, QTc interval, ΔSBP, and ΔDBP with demographic, metabolic, neuropathic, and vascular parameters. Approximate 95% CIs for Spearman’s ρ were calculated using Fisher’s z transformation. Because these bivariate analyses were exploratory and no single primary hypothesis was prespecified for this secondary analysis, multiplicity was addressed using the Benjamini–Hochberg false discovery rate (FDR) procedure rather than retrospectively designating primary outcomes. FDR correction was applied separately within clinically coherent analytic families: continuous between-group comparisons for E:I R–R interval categories, QTc categories, and orthostatic hypotension categories, and the correlation families for E:I R–R interval ratio, QTc, ΔSBP, and ΔDBP. Both nominal *p*-values and FDR-adjusted q-values are reported; q < 0.05 was considered statistically significant after multiplicity correction. For graphical representation, univariable linear regression analyses were performed for the associations of ΔSBP with MNSI and TCNS. Multivariable linear regression models for ΔSBP were adjusted for age, sex, diabetes duration, BMI, and eGFR; the MNSI and TCNS models additionally included HbA1c, with MNSI and TCNS evaluated in separate models. For each multivariable model, the number of observations included (*n*) and the adjusted coefficient of determination (adjusted R^2^) were reported to characterize model sample size and explanatory performance. All tests were two-sided. The sample size was determined for the original study, as previously described. No separate a priori sample size calculation was performed for the present secondary analysis.

## 3. Results

All 109 participants from the previously characterized cohort were included in the present secondary analysis. The baseline demographic, clinical, and metabolic characteristics of the study population have been reported previously [10] (Table 1).

For the present analysis, a pathological E:I R-R interval ratio was identified in 90 patients (82.6%), prolonged QTc in 23 patients (21.1%), and orthostatic hypotension in 40 patients (36.7%).

### 3.1. Association Between E:I R-R Interval Ratio and Clinical and Neuropathic Parameters

No statistically significant between-group differences were observed in age, diabetes duration, body mass index, HbA1c, estimated glomerular filtration rate, ankle–brachial pressure index, peripheral neuropathy severity scores, QTc interval, or orthostatic blood pressure parameters between patients with normal and pathological E:I R–R interval ratios (all *p* > 0.05) (Table 2).

No statistically significant associations were found between the E:I R–R interval ratio and the evaluated demographic, metabolic, vascular, or neuropathic variables, either at the nominal level or after FDR correction (Table 3).

### 3.2. Association Between QT Interval Prolongation and Clinical Parameters

Prolonged QTc was identified in 23 (21.1%) patients, whereas 86 (78.9%) had a normal QTc interval. Participants with prolonged QTc had a nominally higher eGFR than those without QT prolongation (median 88.0 vs. 72.7 mL/min/1.73 m^2^, nominal *p* = 0.025), but this isolated finding did not remain significant after FDR correction (q = 0.301). No other between-group differences were observed (Table 4).

No statistically significant categorical associations were observed between prolonged QTc and pathological E:I R–R interval ratio, orthostatic hypotension, diabetic retinopathy, cardiovascular events, or peripheral arterial disease. Fisher’s exact test was used for comparisons containing an observed cell count < 5; Pearson’s chi-square test was used otherwise (Table 5).

QTc was not significantly correlated with the evaluated demographic, metabolic, vascular, neuropathic, or other autonomic-related continuous measures after FDR correction (all q > 0.05) (Table 6).

### 3.3. Association Between Orthostatic Hypotension and Clinical Parameters

Orthostatic hypotension was identified in 40 patients (36.7%), whereas 69 patients did not meet the diagnostic criteria. No statistically significant differences were observed between patients with and without orthostatic hypotension in age, diabetes duration, BMI, HbA1c, eGFR, neuropathy severity scores, ankle–brachial pressure index, E:I R–R interval ratio, QTc interval, or neutrophil-to-lymphocyte ratio (all *p* > 0.05) (Table 7).

No statistically significant associations were observed between orthostatic hypotension and cardiovascular events (OR 1.48, 95% CI 0.53–4.11; *p* = 0.455) or peripheral arterial disease (OR 1.38, 95% CI 0.52–3.63; *p* = 0.515) (Table 8).

Orthostatic systolic blood pressure change (ΔSBP) showed nominal inverse correlations with HbA1c (ρ = −0.191, nominal *p* = 0.046), TCNS (ρ = −0.406, *p* < 0.001), and MNSI (ρ = −0.534, *p* < 0.001). After Benjamini–Hochberg FDR correction within the ΔSBP correlation family, the associations with TCNS (q < 0.001) and MNSI (q < 0.001) remained significant, whereas the HbA1c association did not (q = 0.123). Univariable linear regression demonstrated inverse associations between ΔSBP and both MNSI and TCNS scores, as illustrated in Figure 1 and Figure 2. Higher peripheral neuropathy severity was associated with more negative ΔSBP values, indicating a greater systolic blood pressure decrease after standing. No ΔDBP correlation remained significant after FDR correction (Table 9).

After adjustment for age, sex, diabetes duration, HbA1c, BMI, and eGFR, the inverse associations between ΔSBP and both MNSI and TCNS remained significant. In contrast, HbA1c was not independently associated with ΔSBP in the adjusted model (Table 10). All three multivariable models included 109 observations. The adjusted R^2^ was 0.233 for the MNSI model, 0.158 for the TCNS model, and −0.009 for the HbA1c model.

## 4. Discussion

The present secondary analysis identified distinct clinical association patterns for the evaluated autonomic-related measures in patients with T2DM. The E:I R–R interval ratio reflects cardiovagal function, the orthostatic blood pressure response reflects sympathetic vasomotor regulation, and QTc is an indirect and non-specific measure influenced by autonomic and non-autonomic factors. These measures should therefore be interpreted as complementary components of an autonomic assessment rather than as individually diagnostic of CAN. No significant clinical correlates were identified for the E:I R–R interval ratio in the present analysis. In contrast, continuous orthostatic systolic blood pressure changes were associated with peripheral neuropathy severity scores, whereas categorical orthostatic hypotension showed no significant associations with the evaluated cardiovascular or neuropathic variables. QTc prolongation showed limited overlap with the other evaluated measures; the isolated nominal difference in eGFR did not remain significant after FDR correction. Collectively, these findings indicate partially distinct patterns of cross-sectional associations among the evaluated measures.

A different pattern was observed when orthostatic blood pressure was analyzed as a categorical versus a continuous measure. Categorical orthostatic hypotension was not associated with cardiovascular events or peripheral arterial disease in this cohort. However, continuous ΔSBP showed inverse associations with peripheral neuropathy severity. This difference should not be interpreted as evidence that categorical orthostatic hypotension and continuous blood pressure responses represent distinct biological phenomena. Rather, dichotomizing a continuous physiological measure may reduce statistical information and power, particularly in a modest sample. Continuous analyses may therefore detect graded associations that are not apparent when the same response is classified using a fixed diagnostic threshold. A more negative ΔSBP was associated with greater peripheral neuropathy severity, as reflected by higher TCNS and MNSI scores. The associations with TCNS and MNSI remained significant after FDR correction and after multivariable adjustment for age, sex, diabetes duration, HbA1c, BMI, and eGFR. By contrast, the nominal univariable association with HbA1c did not survive FDR correction and was not retained in the adjusted model. No ΔDBP correlation remained significant after FDR correction.

Although our study did not establish a diagnosis of cardiovascular autonomic neuropathy using the complete cardiovascular autonomic reflex test battery, the observed association between a more negative ΔSBP and peripheral neuropathy severity is consistent with previous studies reporting an association between peripheral somatic neuropathy and cardiovascular autonomic dysfunction in T2DM. In a cross-sectional study of patients with T2DM and electrophysiological confirmed peripheral neuropathy, cardiovascular autonomic neuropathy severity increased with worsening peripheral neuropathy. Similarly, studies using a complete Ewing test battery have reported associations between cardiovascular autonomic neuropathy and peripheral nerve dysfunction. These studies are not directly comparable with the present analysis; however, they support the biological plausibility of the association observed for continuous orthostatic systolic blood pressure change [11,12].

QTc prolongation showed a limited relationship with the other autonomic and neuropathic abnormalities evaluated in our cohort. Although QTc prolongation has been recognized as a potential manifestation of diabetic autonomic dysfunction, its diagnostic relationship with CAN is not uniform. A meta-analysis of 17 studies including 4584 patients with diabetes reported that QTc prolongation had relatively high specificity but low sensitivity for diabetic autonomic failure, supporting its limited ability to identify the full spectrum of CAN [13].

In contrast to cardiovascular autonomic reflex tests, QTc is not a direct measure of a single autonomic pathway and may be influenced by multiple autonomic and non-autonomic determinants. Therefore, the limited overlap between QTc prolongation and the other evaluated autonomic-related or neuropathic measures does not necessarily contradict its recognized association with CAN. The nominal finding of higher eGFR in participants with prolonged QTc should be regarded as exploratory because it did not survive FDR correction and was not accompanied by a consistent pattern across the other analyses.

Our findings support a heterogeneous view of cardiovascular autonomic dysfunction in T2DM. The three evaluated components did not identify identical clinical profiles. Orthostatic cardiovascular regulation showed robust associations with peripheral neuropathy severity when blood pressure responses were analyzed as continuous physiological variables, whereas the nominal association with glycemic control did not survive multiplicity correction or multivariable adjustment. The categorical diagnosis of orthostatic hypotension had limited discriminatory value in this cohort. The E:I R–R interval ratio and QTc prolongation showed limited overlap with the remaining evaluated clinical measures.

These differences are physiologically plausible because the evaluated measures reflect different aspects of cardiovascular autonomic regulation. Heart rate response to deep breathing predominantly assesses parasympathetic cardiovagal activity, whereas the orthostatic blood pressure response is largely dependent on sympathetic vasomotor regulation. QTc, although influenced by autonomic tone, additionally reflects ventricular repolarization and is affected by several non-autonomic determinants. Consequently, abnormalities in these measures should not necessarily be expected to occur simultaneously or to show identical clinical associations.

From a clinical perspective, these findings emphasize the limitations of relying on a single autonomic-related measure to characterize cardiovascular autonomic involvement in T2DM. Interpretation of cardiovagal, orthostatic, and QTc measures together may provide a more nuanced characterization of autonomic involvement. However, the clinical utility and prognostic relevance of this approach require prospective validation using a complete standardized autonomic test battery.

The main strength of this study is the simultaneous evaluation of several cardiovascular autonomic components within the same well-characterized population of patients with T2DM, together with assessment of peripheral neuropathy, metabolic status, and peripheral vascular involvement. This approach allowed direct comparison of the clinical association patterns of individual autonomic abnormalities.

Several limitations should be acknowledged. This was a prespecified secondary cross-sectional analysis of a single-center cohort of hospitalized patients with T2DM; therefore, temporal sequence and causality cannot be determined, and the findings may not be generalizable to other populations. The sample size was modest, particularly for some subgroups, including participants with prolonged QTc. Although multivariable models were used for the main analyses of continuous ΔSBP, residual confounding cannot be excluded. In particular, cardiovascular, antihypertensive, and other medications that may influence heart rate, blood pressure, and QTc were not systematically recorded. In addition, room temperature was not standardized or recorded. The autonomic assessment did not include the full standardized cardiovascular autonomic reflex test battery. Specifically, the Valsalva maneuver and a standardized heart-rate response-to-standing test were not performed. The use of a single non-age-adjusted threshold for the E:I R–R interval ratio may have overestimated the prevalence of an abnormal cardiovagal response in older participants. Additionally, blood pressure was measured only one minute after standing, which may have limited the detection of delayed OH. QT intervals were measured manually, without standardized independent ECG adjudication, and correction was based on Bazett’s formula, which may overcorrect at higher heart rates and undercorrect at lower heart rates. The large number of exploratory comparisons also creates a risk of type I error; this was addressed by FDR correction within clinically coherent analytic families, but the findings remain hypothesis-generating and require external validation.

## 5. Conclusions

Individual autonomic-related measures showed distinct clinical association patterns in patients with T2DM. Continuous orthostatic systolic blood pressure changes were independently associated with peripheral neuropathy severity, whereas categorical orthostatic hypotension showed no significant clinical associations. The E:I R–R interval ratio and QTc prolongation showed limited overlap with the remaining evaluated measures. None of these individual measures should be interpreted as independently diagnostic of CAN. Larger prospective studies using a complete standardized autonomic test battery are needed to confirm these associations and determine their clinical and prognostic relevance.

## Figures and Tables

**Figure 1:**
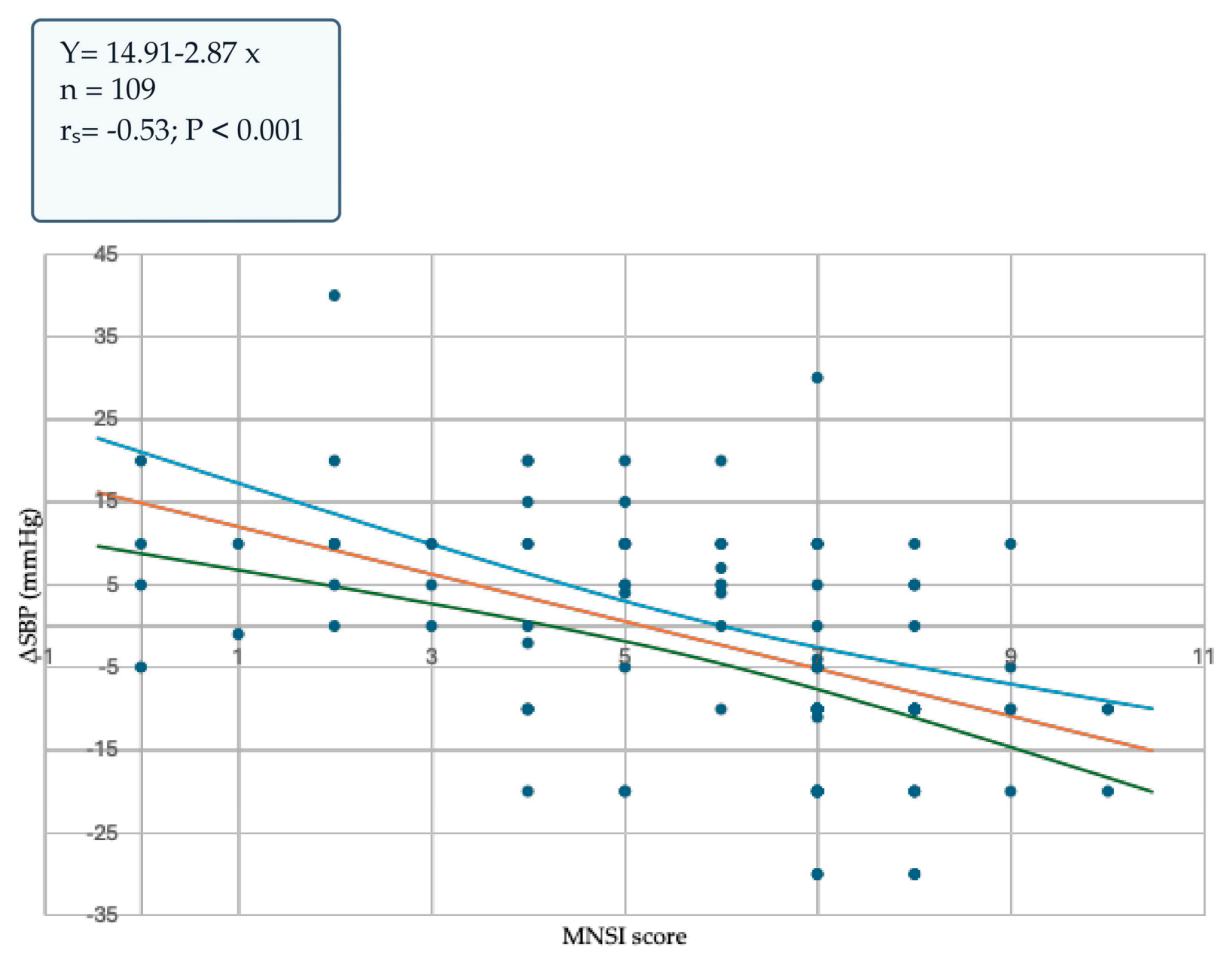
Correlation between MNSI score and orthostatic systolic blood pressure change. Each point represents an individual participant. The central line represents the fitted linear regression line, and the outer lines indicate its 95% confidence intervals.

**Figure 2:**
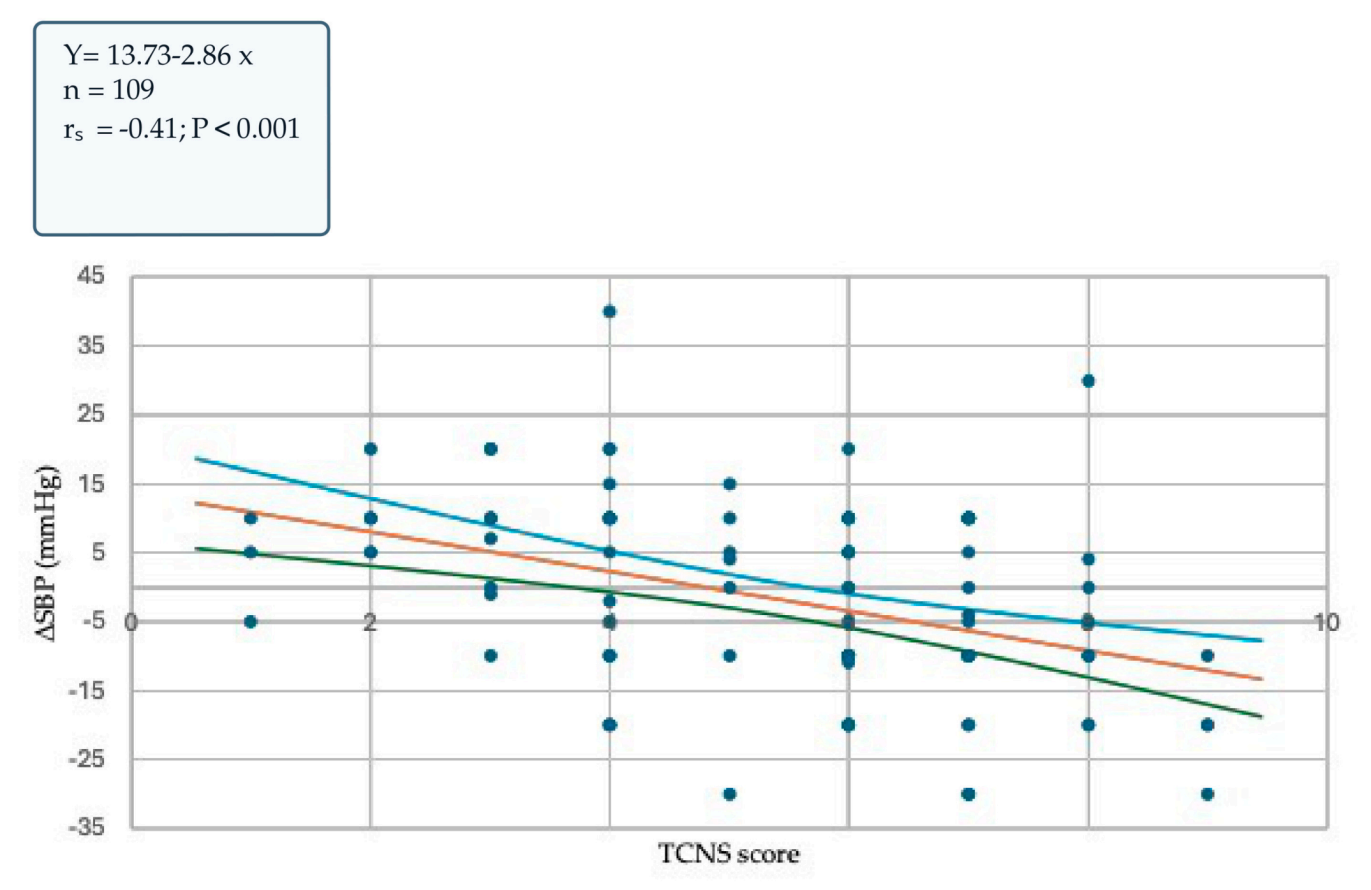
Correlation between TCNS score and orthostatic systolic blood pressure change. Each point represents an individual participant. The central line represents the fitted linear regression line, and the outer lines indicate its 95% confidence intervals.

**Table 1:** Baseline characteristics of the study population.

Parameter	Result
Sex	Men: 56 (51.4%) Women: 53 (48.6%)
Age	65 years [56, 70]
Diabetes duration	12 years [6, 20]
HbA1c	8.49% [7.30, 9.42]
BMI	31.43 ± 5.13 kg/m^2^
eGFR	75 mL/min/1.73 m^2^ [55, 88]
Orthostatic hypotension	OH+: 40 (36.7%) OH−: 69 (63.3%)
Retinopathy	Retinopathy+: 35 (32.1%) Retinopathy−: 74 (67.9%)
PAD	PAD+: 21 (19.3%) PAD−: 88 (80.7%)
CV events	CV+: 18 (16.5%) CV−: 91 (83.5%)
Insulin	Insulin+: 70 (64.2%) Insulin−: 39 (35.8%)
Metformin	Metformin+: 71 (65.1%) Metformin−: 38 (34.9%)
Resting heart rate	72.8 ± 8.7 bpm
MNSI score	7 [4, 7]
TCNS score	6 [4, 7]

Categorical variables are presented as *n* (%). Normally distributed continuous variables are presented as mean ± standard deviation and non-normally distributed variables as median [interquartile range]. HbA1c: hemoglobin A1c; BMI: body mass index; eGFR: estimated glomerular filtration rate; MNSI: Michigan Neuropathy Screening Instrument; TCNS: Toronto Clinical Neuropathy Score; PAD: peripheral artery disease; CV: cardiovascular; OH: orthostatic hypotension.

**Table 2:** Clinical and laboratory characteristics according to E:I R-R interval ratio.

	Normal E:I R-R Interval Ratio *n* = 19	Pathological E:I R-R Interval Ratio *n* = 90	Nominal *p*; FDR q
Diabetes duration (years)	8.00 [4.50, 19.50]	12.00 [8.00, 20.00]	*p* = 0.127; q = 0.331
Orthostatic diastolic blood pressure change (ΔDBP)	0.00 [0.00, 8.50]	0.00 [−10.00, 0.00]	*p* = 0.077; q = 0.331
Orthostatic systolic blood pressure change (ΔSBP)	7.00 [−5.00, 10.00]	−5.00 [−10.00, 10.00]	*p* = 0.067; q = 0.331
HbA1c (%)	8.30 [7.55, 8.93]	8.50 [7.23, 9.73]	*p* = 0.836; q = 0.905
Maximum ABPI	1.01 [1.00, 1.10]	1.10 [1.00, 1.22]	*p* = 0.104; q = 0.331
Minimum ABPI	1.01 [0.99, 1.09]	1.05 [0.97, 1.20]	*p* = 0.277; q = 0.450
BMI (kg/m^2^)	29.98 [28.72, 33.62]	31.74 [27.46, 34.67]	*p* = 0.623; q = 0.737
MNSI score	6.00 [4.00, 6.50]	7.00 [5.00, 7.00]	*p* = 0.122; q = 0.331
Neutrophil-to-lymphocyte ratio	1.94 [1.49, 2.51]	1.83 [1.40, 2.52]	*p* = 0.940; q = 0.940
QTc (ms)	400 [376, 434]	408 [389, 442]	*p* = 0.322; q = 0.465
eGFR (mL/min/1.73 m^2^)	79.00 [66.05, 88.55]	74.88 [53.50, 87.90]	*p* = 0.460; q = 0.598
TCNS score	6.00 [3.50, 6.00]	6.00 [4.00, 7.00]	*p* = 0.185; q = 0.343
Age (years)	60.0 [55.5, 69.0]	65.5 [56.0, 70.0]	*p* = 0.180; q = 0.343

Values are median [interquartile range]. Nominal *p*-values were calculated using the Mann–Whitney U test; q-values are Benjamini–Hochberg FDR-adjusted within this family of comparisons. q < 0.05 denotes statistical significance after multiplicity correction. DBP: diastolic blood pressure; SBP: systolic blood pressure; E:I: expiration-to-inspiration; FDR: false discovery rate; HbA1c: hemoglobin A1c; ABPI: ankle–brachial pressure index; BMI: body mass index; MNSI: Michigan Neuropathy Screening Instrument; QTc: corrected QT interval; eGFR: estimated glomerular filtration rate; TCNS: Toronto Clinical Neuropathy Score.

**Table 3:** Correlations between the E:I R-R interval ratio and clinical parameters.

	Spearman’s ρ (95% CI)	Nominal *p*; FDR q
Maximum ankle–brachial pressure index (Max ABPI)	−0.096 (−0.279 to 0.094)	*p* = 0.321; q = 0.735
Minimum ankle–brachial pressure index (Min ABPI)	−0.071 (−0.256 to 0.118)	*p* = 0.462; q = 0.735
Diabetes duration (years)	−0.087 (−0.270 to 0.103)	*p* = 0.371; q = 0.735
Orthostatic diastolic blood pressure change (ΔDBP)	0.108 (−0.082 to 0.290)	*p* = 0.263; q = 0.735
TCNS score	−0.029 (−0.216 to 0.160)	*p* = 0.767; q = 0.920
Age (years)	−0.120 (−0.301 to 0.070)	*p* = 0.213; q = 0.735
QTc interval (ms)	−0.004 (−0.192 to 0.184)	*p* = 0.964; q = 0.993
BMI (kg/m^2^)	−0.058 (−0.244 to 0.131)	*p* = 0.547; q = 0.735
HbA1c (%)	0.058 (−0.132 to 0.243)	*p* = 0.551; q = 0.735
MNSI score	−0.001 (−0.189 to 0.187)	*p* = 0.993; q = 0.993
Neutrophil-to-lymphocyte ratio	−0.155 (−0.333 to 0.034)	*p* = 0.107; q = 0.735
Orthostatic systolic blood pressure change (ΔSBP)	0.088 (−0.102 to 0.271)	*p* = 0.365; q = 0.735

Values are Spearman’s rank correlation coefficients (ρ) with approximate 95% confidence intervals. Nominal *p*-values and Benjamini–Hochberg FDR-adjusted q-values are reported for the E:I R–R interval correlation family; q < 0.05 denotes statistical significance after multiplicity correction. DBP: diastolic blood pressure; SBP: systolic blood pressure; TCNS: Toronto Clinical Neuropathy Score; E:I: expiration-to-inspiration; CI: confidence interval; FDR: false discovery rate; HbA1c: hemoglobin A1c; ABPI: ankle–brachial pressure index; BMI: body mass index; MNSI: Michigan Neuropathy Screening Instrument; QTc: corrected QT interval.

**Table 4:** Clinical and laboratory characteristics according to QTc interval.

	Normal QTc *n* = 86	Prolonged QTc *n* = 23	Nominal *p*; FDR q
Orthostatic diastolic blood pressure change (ΔDBP)	0.00 [−10.00, 0.00]	0.00 [−2.50, 10.00]	*p* = 0.287; q = 0.748
Orthostatic systolic blood pressure change (ΔSBP)	−3.00 [−10.00, 10.00]	0.00 [−10.00, 10.00]	*p* = 0.689; q = 0.752
Diabetes duration (years)	12.00 [6.50, 20.00]	12.00 [3.00, 20.00]	*p* = 0.331; q = 0.748
HbA1c (%)	8.50 [7.31, 9.73]	8.20 [7.10, 8.75]	*p* = 0.314; q = 0.748
Minimum ABPI	1.03 [0.97, 1.20]	1.06 [1.00, 1.14]	*p* = 0.624; q = 0.748
Maximum ABPI	1.10 [0.99, 1.22]	1.10 [1.01, 1.15]	*p* = 0.838; q = 0.838
BMI (kg/m^2^)	31.23 [27.56, 34.24]	32.30 [27.46, 35.51]	*p* = 0.438; q = 0.748
MNSI score	7.00 [5.00, 7.00]	6.00 [4.00, 7.00]	*p* = 0.473; q = 0.748
E:I R–R interval ratio	1.10 [0.93, 1.16]	1.07 [0.91, 1.15]	*p* = 0.565; q = 0.748
eGFR (mL/min/1.73 m^2^)	72.74 [55.00, 84.62]	88.00 [63.96, 90.05]	*p* = 0.025; q = 0.301
TCNS score	6.00 [4.00, 7.00]	5.00 [4.00, 7.00]	*p* = 0.523; q = 0.748
Age (years)	66.0 [56.0, 70.0]	61.0 [55.0, 67.0]	*p* = 0.122; q = 0.731

Values are median [interquartile range]. Nominal *p*-values were calculated using the Mann–Whitney U test; q-values are Benjamini–Hochberg FDR-adjusted within this family of comparisons. q < 0.05 denotes statistical significance after multiplicity correction. DBP: diastolic blood pressure; SBP: systolic blood pressure; HbA1c: hemoglobin A1c; ABPI: ankle–brachial pressure index; BMI: body mass index; FDR: false discovery rate; MNSI: Michigan Neuropathy Screening Instrument; QTc: corrected QT interval; E:I R–R interval ratio: heart rate response to deep breathing; eGFR: estimated glomerular filtration rate; TCNS: Toronto Clinical Neuropathy Score.

**Table 5:** Associations of prolonged QTc with autonomic abnormalities and diabetes complications.

Variable	Prolonged QTc in Variable+ vs. Variable−, *n*/N (%)	OR (95% CI)	*p*-Value (Test)
Pathological E:I R–R interval ratio	20/90 (22.2%) vs. 3/19 (15.8%)	1.52 (0.40–5.76)	0.759 (Fisher exact)
Orthostatic hypotension	9/40 (22.5%) vs. 14/69 (20.3%)	1.14 (0.44–2.94)	0.785 (χ^2^)
Diabetic retinopathy	6/35 (17.1%) vs. 17/74 (23.0%)	0.69 (0.25–1.95)	0.486 (χ^2^)
Cardiovascular events	2/18 (11.1%) vs. 21/91 (23.1%)	0.42 (0.09–1.96)	0.352 (Fisher exact)
PAD	2/21 (9.5%) vs. 21/88 (23.9%)	0.34 (0.07–1.56)	0.233 (Fisher exact)

For each variable, the table shows the proportion with prolonged QTc among participants with versus without the listed characteristic, together with the odds ratio (OR), 95% confidence interval (CI), and the appropriate two-sided test. Fisher’s exact test was used when any observed cell count was <5; otherwise, Pearson’s chi-square test was used. E:I R–R interval ratio: heart rate response to deep breathing; QTc: corrected QT interval; PAD: peripheral arterial disease.

**Table 6:** Correlations between QTc interval and clinical parameters.

Parameter	Spearman’s ρ (95% CI)	Nominal *p*; FDR q
Maximum ankle–brachial pressure index (Max_ABPI)	−0.007 (−0.195 to 0.181)	*p* = 0.941; q = 0.964
Minimum ankle–brachial pressure index (Min_ABPI)	0.022 (−0.167 to 0.209)	*p* = 0.823; q = 0.964
Diabetes duration (years)	−0.129 (−0.309 to 0.061)	*p* = 0.182; q = 0.364
Orthostatic diastolic blood pressure change (ΔDBP)	0.166 (−0.023 to 0.343)	*p* = 0.084; q = 0.364
TCNS score	−0.132 (−0.312 to 0.058)	*p* = 0.172; q = 0.364
Age (years)	−0.182 (−0.358 to 0.006)	*p* = 0.058; q = 0.364
E:I R-R interval ratio	−0.004 (−0.192 to 0.184)	*p* = 0.964; q = 0.964
BMI (kg/m^2^)	0.156 (−0.033 to 0.335)	*p* = 0.105; q = 0.364
HbA1c (%)	−0.030 (−0.217 to 0.159)	*p* = 0.757; q = 0.964
MNSI score	−0.065 (−0.250 to 0.124)	*p* = 0.499; q = 0.833
Neutrophil-to-lymphocyte ratio	0.139 (−0.050 to 0.319)	*p* = 0.149; q = 0.364
Orthostatic systolic blood pressure change (ΔSBP)	0.057 (−0.132 to 0.243)	*p* = 0.556; q = 0.833

Values are Spearman’s rank correlation coefficients (ρ) with approximate 95% confidence intervals. Nominal *p*-values and Benjamini–Hochberg FDR-adjusted q-values are reported for the QTc correlation family; q < 0.05 denotes statistical significance after multiplicity correction. CI: confidence interval; FDR: false discovery rate; DBP: diastolic blood pressure; SBP: systolic blood pressure; TCNS: Toronto Clinical Neuropathy Score; HbA1c: hemoglobin A1c; ABPI: ankle–brachial pressure index; BMI: body mass index; MNSI: Michigan Neuropathy Screening Instrument; QTc: corrected QT interval.

**Table 7:** Clinical and laboratory characteristics according to orthostatic hypotension status.

	Without OH *n* = 69	With OH *n* = 40	Nominal p; FDR q
Diabetes duration (years)	12.00 [6.00, 20.00]	14.00 [9.00, 20.00]	*p* = 0.502; q = 0.952
HbA1c (%)	8.40 [7.34, 9.29]	8.64 [7.15, 9.61]	*p* = 0.833; q = 0.952
Maximum ABPI	1.10 [1.00, 1.22]	1.08 [1.00, 1.15]	*p* = 0.311; q = 0.952
Minimum ABPI	1.04 [1.00, 1.20]	1.03 [0.96, 1.11]	*p* = 0.190; q = 0.952
BMI (kg/m^2^)	31.21 [27.83, 34.25]	31.85 [27.21, 35.12]	*p* = 0.685; q = 0.952
MNSI score	6.00 [5.00, 7.00]	7.00 [4.00, 8.00]	*p* = 0.771; q = 0.952
Neutrophil-to-lymphocyte ratio	1.76 [1.44, 2.46]	1.94 [1.46, 2.59]	*p* = 0.559; q = 0.952
QTc (ms)	409 [383, 440]	406 [388, 441]	*p* = 0.935; q = 0.952
E:I R-R interval ratio	1.10 [0.93, 1.17]	1.06 [0.92, 1.12]	*p* = 0.261; q = 0.952
eGFR (mL/min/1.73 m^2^)	76.34 [52.93, 88.10]	72.90 [58.10, 87.70]	*p* = 0.756; q = 0.952
TCNS score	6.00 [4.00, 7.00]	6.00 [4.00, 7.00]	*p* = 0.890; q = 0.952
Age (years)	65.0 [56.0, 70.0]	64.5 [56.0, 70.0]	*p* = 0.952; q = 0.952

Values are median [interquartile range]. Nominal *p*-values were calculated using the Mann–Whitney U test; q-values are Benjamini–Hochberg FDR-adjusted within this family of comparisons. q < 0.05 denotes statistical significance after multiplicity correction. HbA1c: hemoglobin A1c; ABPI: ankle–brachial pressure index; BMI: body mass index; MNSI: Michigan Neuropathy Screening Instrument; QTc: corrected QT interval; E:I R–R interval ratio: heart rate response to deep breathing; eGFR: estimated glomerular filtration rate; TCNS: Toronto Clinical Neuropathy Score; OH: orthostatic hypotension; FDR: false discovery rate.

**Table 8:** Associations of orthostatic hypotension with cardiovascular events and PAD.

Variable	Orthostatic Hypotension in Variable+ vs. Variable−, *n*/N (%)	OR (95% CI)	*p*-Value (Test)
Cardiovascular events	8/18 (44.4%) vs. 32/91 (35.2%)	1.48 (0.53–4.11)	0.455 (χ^2^)
PAD	9/21 (42.9%) vs. 31/88 (35.2%)	1.38 (0.52–3.63)	0.515 (χ^2^)

The table shows the proportion with orthostatic hypotension among participants with versus without the listed characteristic, together with the odds ratio (OR), 95% confidence interval (CI), and two-sided Pearson chi-square *p*-value. All observed cell counts in these comparisons were ≥5. PAD: peripheral arterial disease.

**Table 9:** Correlations of orthostatic systolic and diastolic blood pressure changes with clinical parameters.

	ΔSBP		ΔDBP	
	Spearman’s ρ (95% CI)	Nominal *p*; FDR q	Spearman’s ρ (95% CI)	Nominal *p*; FDR q
Age (years)	−0.026 (−0.213 to 0.163)	*p* = 0.788; q = 0.788	−0.145 (−0.324 to 0.044)	*p* = 0.132; q = 0.288
Diabetes duration (years)	−0.156 (−0.334 to 0.033)	*p* = 0.106; q = 0.212	−0.084 (−0.268 to 0.105)	*p* = 0.383; q = 0.438
BMI (kg/m^2^)	−0.075 (−0.259 to 0.115)	*p* = 0.440; q = 0.587	−0.020 (−0.207 to 0.169)	*p* = 0.839; q = 0.839
HbA1c (%)	−0.191 (−0.366 to −0.003)	*p* = 0.046; q = 0.123	−0.141 (−0.320 to 0.049)	*p* = 0.144; q = 0.288
TCNS score	−0.406 (−0.552 to −0.236)	*p* < 0.001; q < 0.001	−0.129 (−0.310 to 0.060)	*p* = 0.180; q = 0.288
MNSI score	−0.534 (−0.656 to −0.384)	*p* < 0.001; q < 0.001	−0.176 (−0.352 to 0.013)	*p* = 0.067; q = 0.288
E:I R–R interval ratio	0.088 (−0.102 to 0.271)	*p* = 0.365; q = 0.583	0.108 (−0.082 to 0.290)	*p* = 0.263; q = 0.350
QTc interval (ms)	0.057 (−0.132 to 0.243)	*p* = 0.556; q = 0.635	0.166 (−0.023 to 0.343)	*p* = 0.084; q = 0.288

Values are Spearman’s rank correlation coefficients (ρ) with approximate 95% confidence intervals. Nominal *p*-values and Benjamini–Hochberg FDR-adjusted q-values are reported separately for the ΔSBP and ΔDBP correlation families; q < 0.05 denotes statistical significance after multiplicity correction. ΔSBP and ΔDBP were calculated as standing minus supine blood pressure; more negative values indicate a greater blood pressure decrease after standing. DBP: diastolic blood pressure; SBP: systolic blood pressure; TCNS: Toronto Clinical Neuropathy Score; HbA1c: hemoglobin A1c; BMI: body mass index; MNSI: Michigan Neuropathy Screening Instrument; QTc: corrected QT interval; E:I: expiration-to-inspiration; CI: confidence interval; FDR: false discovery rate.

**Table 10:** Multivariable linear regression analyses for orthostatic systolic blood pressure change.

	Regression Coefficient (95% CI), mmHg	*p*-Value	n	Adjusted R^2^
MNSI score	−3.00 (−4.03 to −1.96)	<0.001	109	0.233
TCNS score	−3.16 (−4.51 to −1.80)	<0.001	109	0.158
HbA1c, %	−0.14 (−0.41 to 0.13)	0.296	109	−0.009

Each model was adjusted for age, sex, diabetes duration, body mass index, and estimated glomerular filtration rate. The MNSI and TCNS models additionally included HbA1c; MNSI and TCNS were evaluated in separate models. ΔSBP was calculated as standing minus supine systolic blood pressure; more negative values indicate a greater systolic blood pressure decrease after standing. Regression coefficients are unstandardized. CI: confidence interval; HbA1c: glycated hemoglobin; MNSI: Michigan Neuropathy Screening Instrument; TCNS: Toronto Clinical Neuropathy Score. n: number of observations included in the model; adjusted R^2^: adjusted coefficient of determination.

## Data Availability

The data presented in this study are available on request from the corresponding author due to restrictions imposed by the General Data Protection Regulation procedure in the hospital in which study was conducted.
